# Downregulation of SOX2 by inhibition of Usp9X induces apoptosis in melanoma

**DOI:** 10.18632/oncotarget.27869

**Published:** 2021-02-02

**Authors:** Harish Potu, Malathi Kandarpa, Luke F. Peterson, Alison Durham, Nicholas J. Donato, Moshe Talpaz

**Affiliations:** ^1^Department of Internal Medicine/Division of Hematology/Oncology, University of Michigan, School of Medicine and Comprehensive Cancer Center, Ann Arbor, MI 48109, USA; ^2^Department of Dermatology, University of Michigan School of Medicine, Ann Arbor, MI 48109, USA; ^3^Center for Scientific Review, National Institutes of Health, Bethesda, MD 20892, USA; ^*^These authors jointly supervised this work

**Keywords:** melanoma, deubiquitinase (DUB) enzyme, transcription factors, USP9X, DUB inhibitor

## Abstract

Melanoma tumors driven by BRAF mutations often do not respond to BRAF/MEK/ERK pathway inhibitors currently used in treatment. One documented mechanism of resistance is upregulation of SOX2, a transcription factor that is essential for tumor growth and expansion, particularly in melanoma tumors with BRAF mutations. Targeting transcription factors pharmacologically has been elusive for drug developers, limiting treatment options. Here we show that ubiquitin-specific peptidase 9, X-linked (Usp9x), a deubiquitinase (DUB) enzyme controls SOX2 levels in melanoma. Usp9x knockdown in melanoma increased SOX2 ubiquitination, leading to its depletion, and enhanced apoptotic effects of BRAF inhibitor and MEK inhibitors. Primary metastatic melanoma samples demonstrated moderately elevated Usp9x and SOX2 protein expression compared to tumors without metastatic potential. Usp9x knockdown, as well as inhibition with DUB inhibitor, G9, blocked SOX2 expression, suppressed *in vitro* colony growth, and induced apoptosis of BRAF-mutant melanoma cells. Combined treatment with Usp9x and mutant BRAF inhibitors fully suppressed melanoma growth *in vivo*. Our data demonstrate a novel mechanism for targeting the transcription factor SOX2, leveraging Usp9x inhibition. Thus, development of DUB inhibitors may add to the limited repertoire of current melanoma treatments.

## INTRODUCTION

Recent progress in targeting mutant pathways in metastatic melanoma has led to many improvements in treatment and patient survival. Combination of BRAF inhibitors (BRAFi, vemurafenib) and MEK inhibitors (MEKi, PD0325901) extended median progression-free survival from 7 to 11 months as compared to vemurafenib alone [1]. However, many characteristics of melanoma remain elusive and do not explain why only a small subset of patients respond to BRAF and/or MEK inhibitors and only for a limited duration (6–9 months) [2]. Additional research is needed to define other cellular targets and effective treatment strategies in both newly diagnosed and kinase BRAF and MEK inhibitor-resistant melanoma patients. Several mechanisms for resistance to BRAFi have been described, including many genetic alterations that reactivate MAPK signaling such as NRAS mutations [3], MEK mutations or mutant BRAF amplification [4]. BRAFi and MEKi combination therapy does not prevent acquired resistance, which can emerge via similar genetic mechanisms as arise during monotherapy [5, 6]. Moreover, no clear molecular resistance mechanism has been found in melanomas suggesting that transcriptome or epigenetic alterations may underlie acquired MAPK inhibitor (MAPKi) resistance [7]. Therefore, identification of alternate biological pathways that contribute to resistance may lead to the design of more effective combination therapies [8].

SRY (sex determining region Y)-box 2 (SOX2) is a SOX family transcription factor (TF) and plays a role in developmental regulation [9]. SOX2 is important in the maintenance of pluripotency and self-renewal in embryonic stem cells and induced pluripotent stem cells [10]. A recent report suggests SOX2 as an essential TF for the self-renewal capacity of cancer stem cells implicating SOX2 as an oncogenic TF [11]. Other studies have shown that SOX2 plays a significant role in melanoma progression and cell invasion [12]. In skin, SOX2 is expressed in cutaneous neuroendocrine carcinoma (Merkel cell carcinoma) in addition to other subsets of melanoma. Moreover, SOX2 expression in melanoma patient samples was found to correlate with increased tumor thickness [13]. Therefore, SOX2 is an attractive therapeutic target for melanoma. However, unlike protein kinases, TFs like SOX2 are commonly considered as “undruggable” due to lack of an active pocket or well-defined ligand-binding domain. Inducing the degradation of TFs like a IKZF1 (IKAROS Family Zinc Finger 1) by lenalidomide has led to its approval in the treatment of myeloma [14]. This indicates that promoting the degradation of SOX2 may be an alternative approach to target SOX2. Thus, understanding the mechanisms regulating the stability and degradation of SOX2 in melanoma especially by the ubiquitin-proteasome system might yield some strategies to target this oncogenic factor. A few different E3 ubiquitin ligases for SOX2 have been reported including CDC20 and WWP2 [15, 16]. However, mechanisms of stabilizing SOX2 in melanoma have not been interrogated.

Deubiquitinases (DUBs) are key regulators of cellular protein homeostasis. Many DUBs have been shown to be mutated or overexpressed in cancer, including melanoma [17, 18]. Usp9x deubiquitinates proteins essential in cancer cell signaling and survival, as non-ubiquinated proteins are not targeted for degradation by the proteasome [19]. We previously described Usp9x expression and activity in melanoma [20] and further investigated the role of Usp9x in melanoma growth. To investigate mechanisms underlying BRAFi resistance in melanoma, we initially assessed SOX2 levels in BRAF-mutant melanoma. Vemurafenib and MEKi treatment induced SOX2 in a time-dependent manner in both BRAF- and NRAS-mutant melanoma. We identified SOX2 as a substrate of Usp9x in melanoma and determined that SOX2 escapes proteasomal destruction by Usp9x-mediated deubiquitination. Here we show that Usp9x plays a key role in SOX2 regulation and in melanoma tumorigenicity, particularly in tumors driven by BRAF mutation and dependent on SOX2.

## RESULTS

### SOX2 expression is induced by BRAF and MEK inhibitors

**Figure 1:**
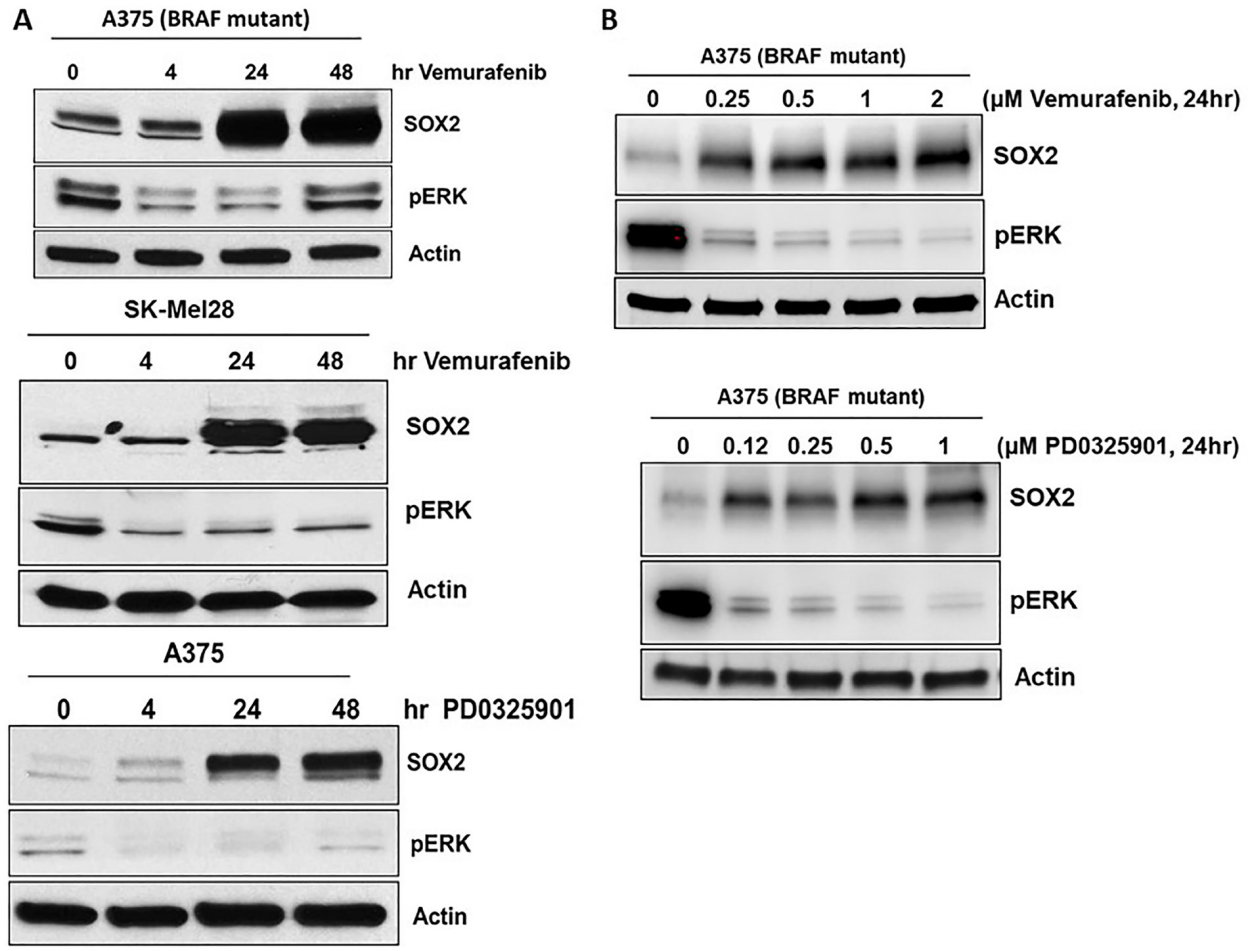
SOX2 expression is induced by BRAF and MEK inhibitors. (**A**) Melanoma cell lines with mutant-BRAF, A375 and SK-Mel28 cells were treated with vemurafenib or PD0325901 for the designated time points. (**B**) Melanoma cell line with mutant-BRAF, A375 cells were treated with vemurafenib or PD0325901 at the designated doses. Immunoblots for SOX2 showed induction as early as 4 hrs and was sustained for 48 hrs. Phospho ERK immunoblot shows activation of MAPK pathways. Actin served as a loading control.

Several mechanisms of acquired resistance to BRAF inhibitors have been described including upregulation of receptor tyrosine kinases [21], serine threonine kinase COT and NRAS [21–23]. We previously noted that Ets-1 TF expression was induced by BRAFi and MEKi in melanoma [20]. Therefore, we further examined the expression of other TFs after treatment with BRAFi and MEKi in melanoma. Analysis of gene expression data (microarray) from a recent publication suggests vemurafenib treatment leads to specific upregulation of SOX2 but not SOX11 and SOX13 (Supplementary Figure 1) [24]. SOX2 induction (mRNA and protein) was observed by mutant BRAF inhibitor vemurafenib treatment in melanoma [25]. We confirm that protein levels of TF SOX2 were induced in mutant BRAF melanoma cell lines, A375 (top) and SK-Mel28 (middle), treated with BRAFi, vemurafenib, and MEKi, PD0325901 (Figure 1A). We also show that protein levels of TF SOX2 were induced dose dependent in mutant BRAF melanoma cell lines, A375, treated with BRAFi, vemurafenib, and MEKi, PD0325901 (Figure 1B). Pharmacological inhibition of BRAF with vemurafenib and MEK with PD0325901 markedly reduced basal phospho-ERK (pERK) in melanoma cells while increasing SOX2, and this induction was specific to the inhibitor treatment as it was time dependent (Figure 1).

### SOX2 levels are controlled by Usp9x-mediated deubiquitination

**Figure 2:**
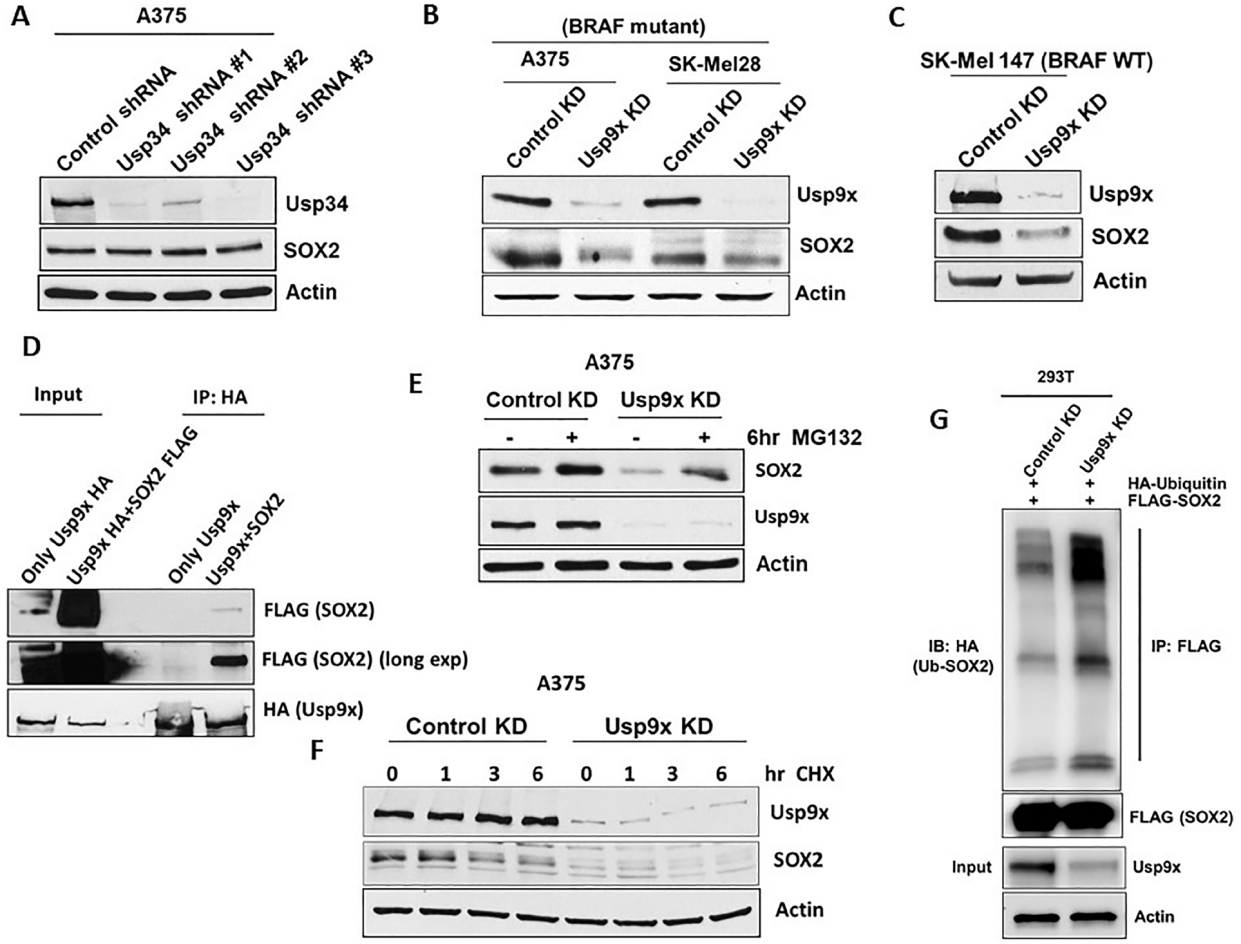
Usp9x deubiquitinates SOX2 and regulates its degradation. Deubiquitinase or transcription factor knock-down (KD) or overexpression in melanoma cell lines was achieved using lentiviral constructs. (**A**) After KD of Usp34 with three different shRNA constructs, immunoblot for Usp34 and SOX2 is shown in mutant-BRAF melanoma cell line. (**B**) KD of Usp9x and immunoblot for the proteins indicated in mutant-BRAF A375melanoma cell lines. (**C**) Immunoblot for the proteins indicated in NRAS mutant melanoma cell lines with control or Usp9x KD. (**D**) Exogenously expressed HA-Usp9x (full-length) was co-expressed with FLAG-SOX2 in HEK293T cells. HA (Usp9x) immunoprecipitation was followed by immunoblotting of FLAG-SOX2, total lysate was used as a control. (**E**) Immunoblot for Usp9x, SOX2, in control and Usp9x KD A375 BRAF-mutant cells treated ± MG132 for 6 h (10 μM). (**F**) Immunoblot for Usp9x and SOX2 after Usp9x KD in A375 BRAF-mutant cells treated with cycloheximide for 6 hrs. (**G**) HEK293T cells exogenously expressing FLAG-SOX2 and HA-ubiquitin were subjected to control or Usp9x KD and FLAG immunoprecipitation was followed by HA blotting to detect Ub-SOX2 levels. Immunoblot for FLAG (SOX2) in the pulldowns (top) and input lysate (Usp9x and actin, bottom) is shown. Actin served as a loading control wherever necessary.

SOX2 protein levels have been shown to be regulated by E3 ligases, CDC20 [15] and WWP2 [16] which promote ubiquitination and degradation of SOX2. In addition, SOX2 interacts with deubiquitinases Usp34 and Usp9x which can lead to SOX2 stabilization in medulloblastoma cells [26] and was also shown in embryonic stem cells [27]. It was shown that SOX2 levels are regulated by Usp9x in osteosarcomas [28]. However, in melanoma SOX2 regulation by Usp9x has not been previously reported. Therefore, we examined the DUBs that regulate SOX2 in melanoma cells. We first knocked down (KD) Usp9x and Usp34 by lentiviral shRNA expression. Usp9x KD but not Usp34 KD (Figure 2A) reduced SOX2 levels in both BRAF mutant A375, SK-Mel28 (Figure 2B), and NRAS-mutant SK-Mel147 (Figure 2C) melanoma cell lines. We next assessed if Usp9x and SOX2 interact. Association between Usp9x and SOX2 was determined by immunoprecipitation and immunoblotting (Figure 2D). We noticed that proteasome inhibitors MG132 and bortezomib can transiently increase SOX2 levels in melanoma cells, suggesting proteasomal degradation of SOX2 (Supplementary Figure 2A). We further demonstrated that Usp9x KD led to SOX2 proteasomal degradation in melanoma. MG132 treatment reversed the reduction of SOX2 by Usp9x KD, indicating that ubiquitin proteasome pathway plays a role in controlling SOX2 levels (Figure 2E). Furthermore, we found that Usp9x KD reduced the half-life of SOX2 in the presence of cycloheximide from 6 to 4 h (Figure 2F), suggesting that in the absence of new protein translation, Usp9x can control pre-existing SOX2 levels. We next tested the ability of Usp9x to deubiquitinate SOX2. Immunoprecipitation of FLAG-SOX2 from HEK293T cells also expressing HA-ubiquitin, we show that Usp9x KD increased the levels of ubiquitinated SOX2 in the cells (Figure 2G). We next co-expressed FLAG-SOX2 with wild type HA-ubiquitin or mutant ubiquitin that preferentially forms Lys-48 or Lys-63-linked chains. Immunoprecipitation of the ubiquitinated FLAG-SOX2 revealed preferential increase of the ubiquitinated pattern using K63-linked HA-ubiquitination (Supplementary Figure 2C). To further assess regulation of SOX2 by deubiquitination in melanoma, we examined the activity of our recently described DUB inhibitor, G9 [20, 27] (Supplementary Figure 2B top). G9 inhibits Usp9x activity *in vitro* and *in vivo,* and leads to tumor inhibition and regression [20, 29]. We showed that similar to Usp9x KD (Figure 2E), G9 treatment also decreased SOX2 levels in BRAF-mutant melanoma cells and MG132 treatment reversed the reduction of SOX2 by G9 (Supplementary Figure 2B bottom). Considered together, our results indicate that SOX2 is a polyubiquitinated protein with an unexpectedly high turnover rate in melanoma cells which can be controlled by the deubiquitinase Usp9X.

### Usp9x regulates apoptotic response to mutant BRAF inhibition in melanoma

**Figure 3:**
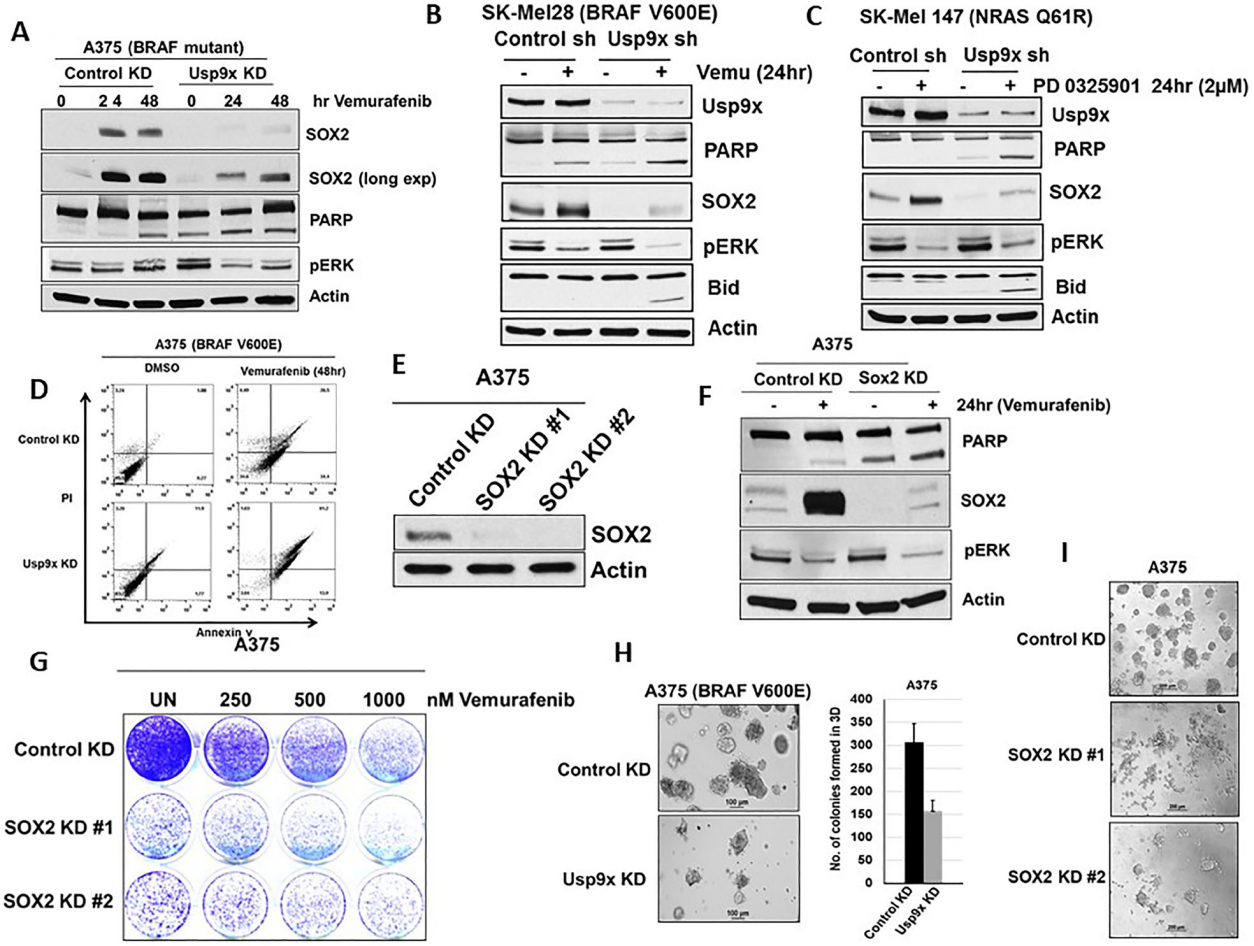
Blocking SOX2 induction increases apoptosis by MAPK pathway inhibitors. Vemurafenib and PD0325901 treatment in melanoma cells with or without Usp9x KD. (**A**) A375 BRAF mutant cells followed by immunoblot for the proteins indicated. (**B**) SK-Mel28 BRAF mutant cells followed by immunoblot for the proteins indicated. Cleavage of PARP and Bid induction was indicated apoptosis in melanoma. (**C**) SK-Mel147 BRAF mutant cells followed by immunoblot for the proteins indicated in NRAS mutant (SK-Mel147) cells with or without Usp9x KD treated with MEKi inhibitors as indicated. Cleavage of PARP and Bid induction was indicated apoptosis in NRAS mutant melanoma. Actin served as a loading control. (**D**) Annexin V assessment in control and Usp9x KD BRAF-mutant (A375) cells treated with vemurafenib (1 μM) for 48 hours as indicated. (**E**) SOX2 KD confirmed by immunoblotting with two different shRNAs. (**F**) Immunoblot for the proteins indicated in BRAF mutant (A375) cells with or without SOX2 KD treated with vemurafenib as indicated. (**G**) Images of colony growth (detected by crystal violet staining) in BRAF-mutant A375 cells with or without vemurafenib as indicated in control and SOX2 KD cells after 21 days in standard 2D culture. (**H**) Phase-contrast images of BRAF-mutant A375 cells with or without Usp9x KD grown in 3D (matrigel) for 7 days (left) quantification of colony growth (right). (**I**) Phase-contrast images of BRAF-mutant A375 cells with or without SOX2 KD grown in 3D (matrigel) for 7 days.

Since SOX2 TF plays a role in cell growth and proliferation, we next wanted to know whether Usp9x KD can regulate the cellular response to vemurafenib via lowering SOX2 and enhancing apoptosis. Usp9x KD blocked the induction of SOX2 by vemurafenib or MEKi treatment in melanoma cell lines with mutant BRAF, A375 (Figure 3A), SK-Mel28 (Figure 3B) and wild type BRAF, SK-Mel147 (Figure 3C). Furthermore, Usp9x KD increased PARP and BID cleavage, a hallmark of apoptosis induction in all three cell lines (Figure 3A–3C). Usp9x KD facilitated the induction and level of apoptosis by vemurafenib in BRAF-mutant cells and MEK inhibitors in BRAF WT/NRAS-mutant cells. These effects correlated with additive cell growth inhibition (Supplementary Figure 3A) and apoptosis induction as measured by Annexin V staining compared with kinase inhibition alone (Figure 3D). The correlation between reduction of SOX2 signaling via Usp9x KD and apoptotic response to BRAF inhibitors led us to further investigate if SOX2 downregulation alone is sufficient for the observed response. Previous studies have shown that SOX2 KD suppresses cell growth and induces apoptosis in melanoma [11]. Therefore, we investigated the effects of SOX2 KD on melanoma cell viability and death. Using two different shRNAs, SOX2 KD with shRNA was confirmed by immunoblotting (Figure 3E). SOX2 KD also induced apoptosis (cleavage of PARP) alone and co-operated with vemurafenib in mutant BRAF cell line (A375) (Figure 3F). SOX2 KD alone inhibited colony growth and cooperated with vemurafenib in reducing number of colonies in 2D standard culture (Figure 3G). To further assess long-term consequences of Usp9x and SOX2 inhibition, we analyzed the ability of melanoma cells to form colonies in 3-dimensional (3D) growth conditions in matrigel that more closely recapitulates tumor growth *in vivo*. Upon Usp9x KD, colony growth in 3D culture was blocked in both BRAF (A375) (Figure 3H) and NRAS (WM1366)-mutant melanoma cells (Supplementary Figure 3B). In addition, treatment with Usp9x inhibitor G9 for 3 days also inhibited melanoma growth in matrigel-3D cultures (Supplementary Figure 3C). We further examined long-term consequences of SOX2 KD. We found that SOX2 KD blocked colony growth in melanoma (3D culture) (Figure 3I). Hence, our data suggest that Usp9x depletion leads to robust kinase inhibitor induced apoptosis of melanoma cells which is most likely mediated by decreased SOX2 protein.

### Level of SOX2 expression correlates with sensitivity to DUB inhibition

**Figure 4:**
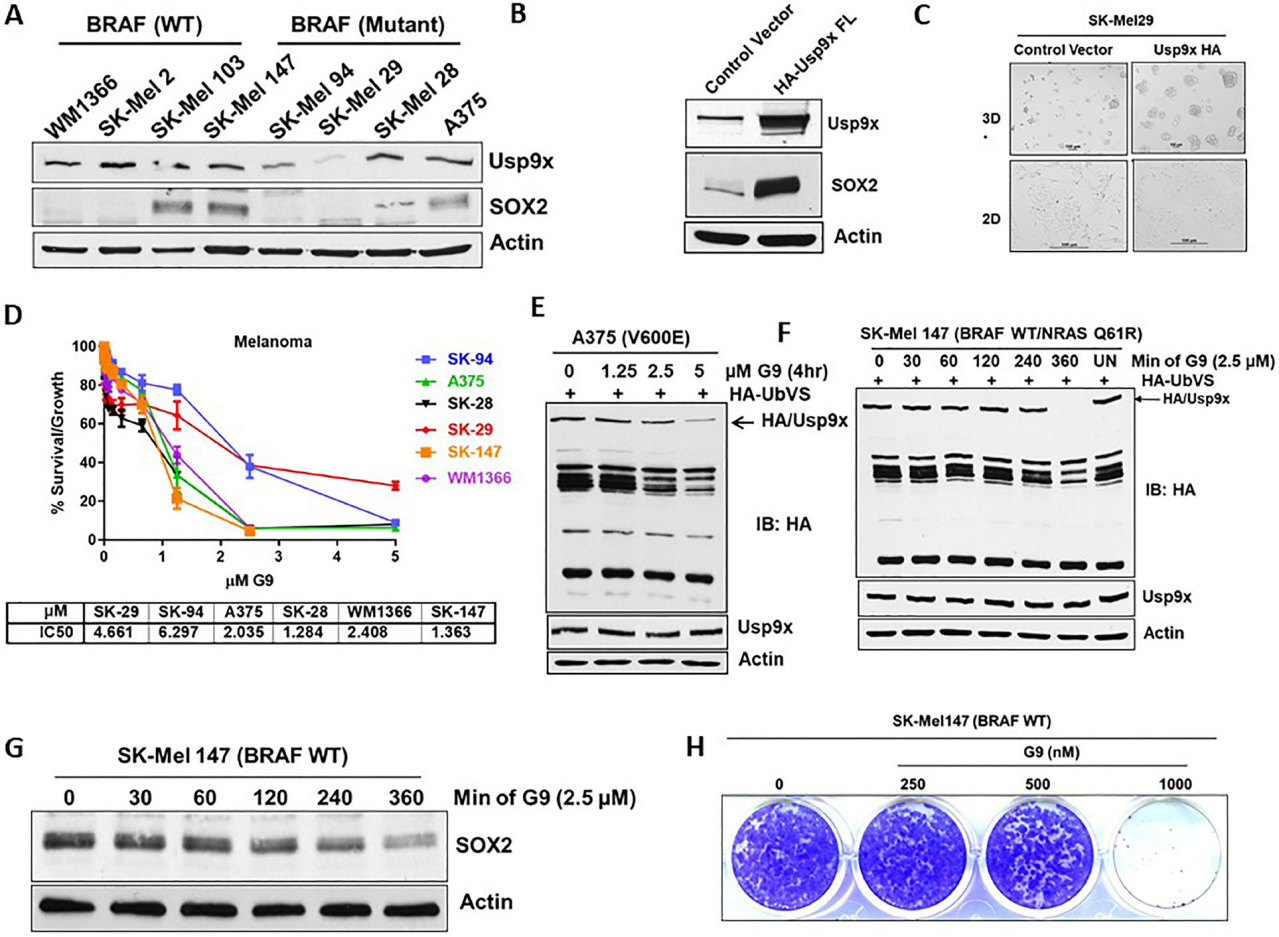
Melanoma cells that express Usp9x and SOX2 are vulnerable to G9. (**A**) Immunoblot for expression of indicated proteins in BRAF- and NRAS-mutant cells. (**B**) Immunoblot for SK-Mel29 cells overexpressing HA-Usp9x. (**C**) Phase-contrast images of SK-Mel29 cells expressing HA-Usp9x grown on matrigel for 7 days. (**D**) Melanoma BRAF and NRAS mutant cell lines were plated and treated with the indicated dose of G9 DUB inhibitor for 72 h and cell proliferation was assessed by MTT assay. The results represent the average +/– S.D. of 4 replicates. IC50 values are indicated. (**E**) A375 mutant melanoma cells were treated with indicated dose of G9 for indicated time and lysates were incubated with HA-UbVS and DUB activity was assessed by HA blotting (top). The HA-labeled Usp9x is detected by Usp9x (total) immunoblotting (below) (**F**) NRAS mutant melanoma cells were treated with indicated dose of G9 and lysates were incubated with HA-UbVS and DUB activity was assessed by HA blotting (top). The HA-labeled Usp9x is detected by Usp9x (total) immunoblotting (below). (**G**) Immunoblot for SOX2 proteins in NRAS-mutant cells treated with G9 as indicated. (**H**) Images of colony growth (detected by crystal violet staining) in NRAS-mutant SK-Mel147 cells were treated with G9 for 21 days (fresh drug added every three days) in standard 2D culture. Actin served as a loading control wherever necessary.

We hypothesized that if Usp9x and SOX2 play an essential role in melanoma cell growth, SOX2 and Usp9x should be co-overexpressed in melanoma. We examined Usp9x and SOX2 expression levels in a panel of BRAF- and NRAS-mutant melanoma cell lines. Although, some cells did not express SOX2 but expressed Usp9x, other cells that lacked Usp9x expressed lower amounts of SOX2 as in SK-Mel29 (Figure 4A). Exogenous overexpression of Usp9x in SK-Mel29 cells lead to upregulation of SOX2 (Figure 4B) and as expected, increased 3D tumor growth (Figure 4C). We have previously shown that G9 inhibited Usp9x activity *in vitro* and *in vivo* and led to melanoma tumor inhibition and regression [20, 29]. Dose response of a panel of melanoma cell lines to G9, showed that BRAF mutant melanoma cells were sensitive to G9, and the degree of sensitivity correlated with Usp9x and SOX2 expression (Figure 4D). G9 inhibits Usp9x activity in A375 BRAF-mutant cells (Figure 4E) and in NRAS-mutant SK-Mel 147 cells (Figure 4F) with corresponding reduction in the levels of SOX2 within 6 hr (Figure 4G). G9 also blocked colony growth in NRAS-mutant melanoma cells (Figure 4H). We hypothesized that Usp9x and SOX2 axis may play a broader role in other cancers. SOX2 promotes lineage plasticity in prostate cancers lacking p53 and RB1, and controls self-renewal in neuroendocrine prostate cancer (NEPC) [30, 31]. Usp9x deubiquitinates and stabilizes the TF, ERG, in prostate, and we previously published that DUB inhibitor (WP1130) has anti-tumor activity in ERG fusion driven prostate cancer [16, 20, 32]. We further tested the Usp9x inhibitor G9 in ERG positive (VCap), ERG negative (DU-145) prostate cell lines and a NEPC line (H660). We found that NEPC H660 cells, were more sensitive among prostate cell lines (Supplementary Figure 4A). G9 treatment led to degradation of SOX2 in a dose dependent manner (top) along with inhibition of Usp9x DUB activity in ERG negative (PC-3) prostate cell lines (Supplementary Figure 4B). G9 effectively inhibited the formation of NEPC H660 colonies in 3-D cultures (Supplementary Figure 4C, top reduced SOX2 protein along with the neuro endocrine marker synaptophysin, which is a biomarker for NEPC diagnosis [44] (Supplementary Figure 4C, bottom). We also analyzed data from cBioPortal [33] and found overexpression of Usp9x in NEPC (Supplementary Figure 4D). Collectively, these results suggest that not only in melanoma, but also in prostate cancer SOX2 expression is controlled via Usp9x.

### Usp9x and SOX2 co-expression in primary metastatic melanoma

**Figure 5:**
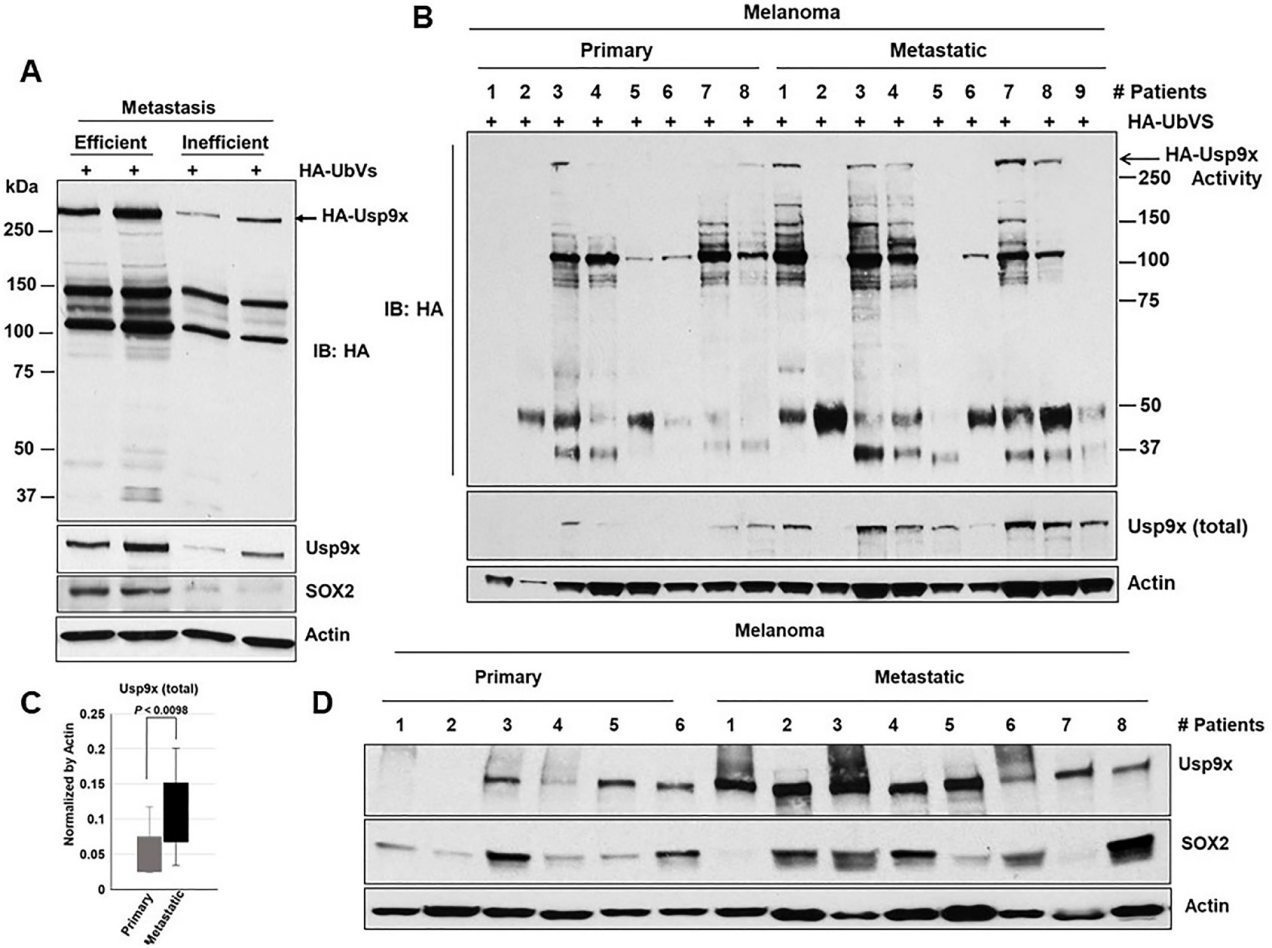
Usp9x and SOX2 are co-expressed in metastatic melanoma. Tumor tissue from primary human melanoma explants established in NSG mice [33]. (**A**) Tumor tissue lysates were incubated with HA-UbVS and DUB activity was assessed by HA blotting (top). The HA-labeled Usp9x is detected by Usp9x (total) immunoblotting (below). (**B**). Primary and metastatic melanoma patient tumors lysates were incubated with HA-UbVS and DUB activity was assessed by HA blotting (top). The HA-labeled Usp9x is detected by Usp9x (total) immunoblotting (below). (**C**) Usp9x protein (total) levels (from B) were quantified by densitometry (ImageJ software). (**D**) Immunoblot for Usp9x, SOX2 in primary and metastatic melanoma patient tumors and Actin served as a loading control wherever necessary.

Correlation of expression of SOX2 and Usp9x is evident in cell line models so we next interrogated this in human primary melanoma cells and metastatic tumors. We used 4 different human tumors explanted in mice, 2 were BRAF mutant and 2 NRAS mutant. It was recently reported that the metastatic activity of these primary tumors in NSG mice mirrors the clinical outcome in patients [34]. The cells were injected in NSG mice and the rate of metastasis was assessed by the percentage of mice with macrometastases (Supplementary Figure 5). We show that Usp9x activity and protein expression as well as SOX2 protein (bottom) expression are elevated in efficient metastatic tumors when compared to those with inefficient metastatic activity (Figure 5A). Usp9x expression was found to be moderately high in metastatic patients as reported in 19 patient-derived primary melanoma cells [20]. We also examined expression levels of Usp9x and SOX2 in fresh tumor tissue from melanoma patients primary or metastatic sites. DUB activity (Usp9x) assays (Figure 5B), measurement (Figure 5C) and immunoblotting (Figure 5D). Suggested that Usp9x activity and expression were elevated in metastatic as compared to primary tumor and correlated with elevated SOX2 levels. Together, these results suggest that Usp9x is overexpressed in metastatic melanoma and might contribute to stabilization of SOX2.

### Usp9x inhibition in combination with vemurafenib blocks *in vivo* tumor growth

**Figure 6:**
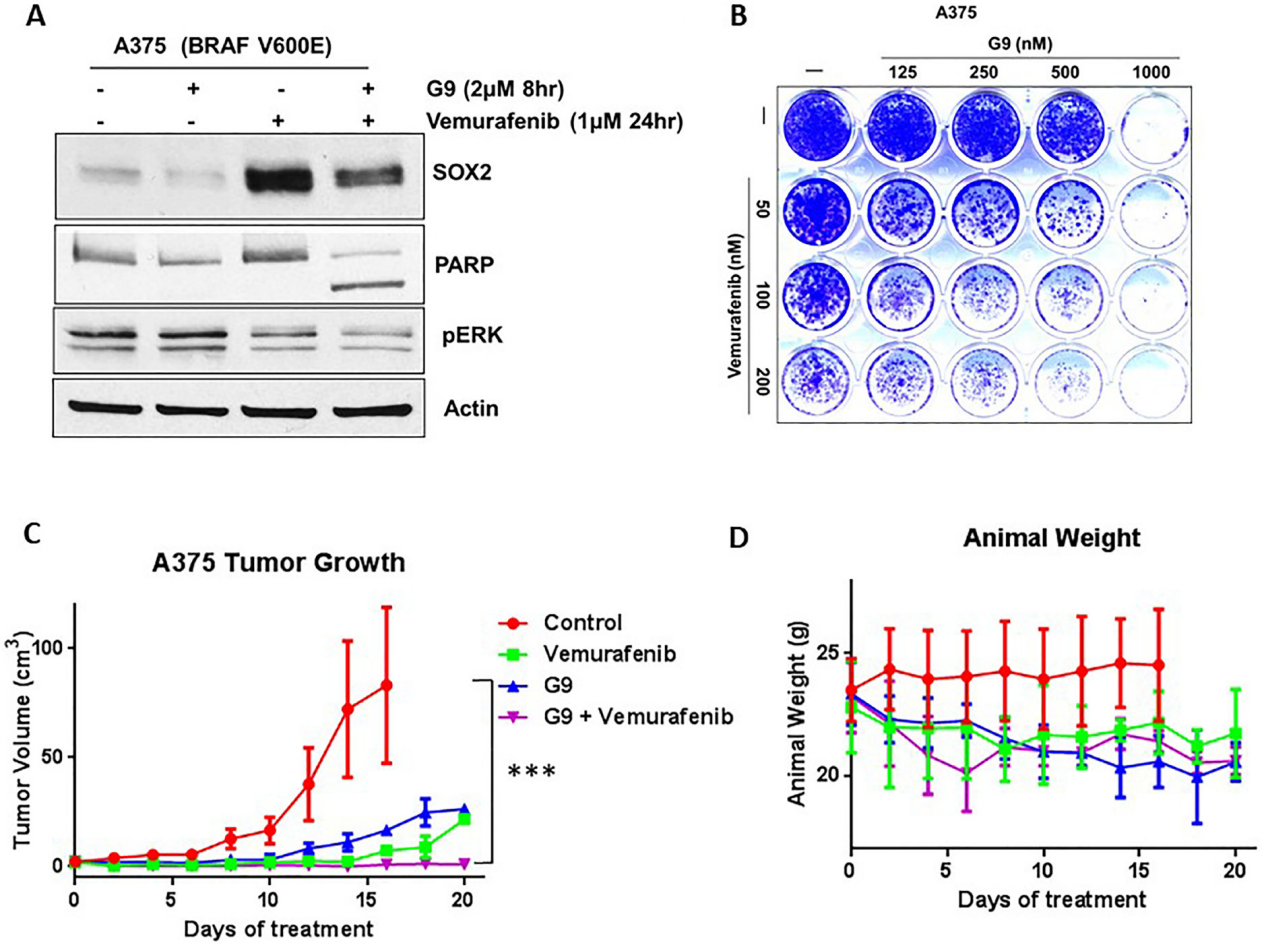
G9 cooperates with inhibition of mutant BRAF to inhibit tumor growth *in vivo*. (**A**) Vemurafenib, G9 and combination treatment in *in vitro* and *in vivo* in melanoma cells. A375 BRAF mutant cells were treated with vemurafenib, G9, or the combination before cell lysates were examined for indicated proteins. (**B**) Images of colony growth (detected by crystal violet staining) in BRAF-mutant A375 cells, which were treated with vemurafenib, G9, or the combination for 21 days (fresh drug was added every three days) in standard 2D culture. (**C**) NSG mice with A375 BRAF mutant melanoma tumors were treated with 25 mg/kg vemurafenib daily, 15 mg/kg G9 every other day, or both for 3 weeks by i.p injection, and tumor dimensions were measured. For each treatment four individual mice were used. (**D**) Animal weight was recorded through the treatment interval. Actin served as a loading control wherever necessary. ^***^ *P* < 0.001. The statistically significant values were measured by Mann–Whitney *U* test for comparisons of control vs G9 (*p* value 0.1308), control vs vemurafenib (*p* value 0.0097), and control vs combination (G9+vemurafenib) (*p* value < 0.0001).

Although inhibitors of mutant BRAF, such as vemurafenib [35], have led to remarkable responses in patients with melanoma, the duration of response is short-lived at 6–9 months [36]. We have previously reported that G9 can overcome acquired resistance to vemurafenib via DUB inhibition [20]. First, we noted that vemurafenib induced SOX2 protein in melanoma cells (Figure 1). We next examined combination treatment with vemurafenib and G9 in melanoma cells. The combination treatment significantly increased apoptosis as measured by PARP cleavage accompanied by reduced SOX2 compared to either agent alone (Figure 6A). Combination treatment also significantly reduced colony growth in 2D tissue cultures of A375 cells after 3 weeks compared to either agent alone (Figure 6B). We further examined whether combined inhibition of mutant BRAF and DUBs would co-operate to reduce tumor burden in xenograft models of melanoma. Mice inoculated with BRAF-mutant A375 cells were treated with G9, vemurafenib or their combination, and tumor growth was assessed over a 3-week treatment period. Both G9 and vemurafenib alone reduced tumor growth (Figure 6C), but refractory tumor cells emerged by the end of the treatment period. Combined G9 and vemurafenib treatment completely blocked tumor growth measured *in vivo* (Figure 6C), while body weight was not significantly affected, suggesting lack of toxicity (Figure 6D) of the combination. DUB activity levels were significantly reduced in two excised tumors from G9-treated mice that demonstrated robust response to G9 (Supplementary Figure 6). These results suggest that DUB inhibition can enhance the antitumor activity of kinase inhibitors to suppress tumor growth in melanoma.

## DISCUSSION

Melanoma patients with mutant BRAF expressing tumors can be treated with BRAF and MEK kinase inhibitors with substantial clinical activity but is limited in duration. This suggests that a thorough understanding of the molecular and cellular pathways activated by mutant BRAF and pathways leading to the resistance to BRAF inhibition may provide insight into a means of improving the therapeutic effectiveness of the kinase inhibitors. We demonstrate that upregulation of SOX2 upon RAS/BRAF/ERK pathway inhibition could be an adaptive resistance signal. Thus, inducing degradation of SOX2 via Usp9x inhibition is a promising therapeutic approach to overcome this adaptive resistance in melanoma. Such adaptive resistance mechanisms are suggested in many cancer cells as well as in induced pluripotent cancer cells (iPCCs) which are highly resistant to the RAS/BRAF/ERK kinase pathway inhibitors vemurafenib and trametinib [37]. SOX2 is an important TF for the reprogramming of iPCCs, and the development of adaptive resistance in these cells [38]. SOX2 not only plays a vital role in cancer cell stemness, but also in invasion and metastatic potential [39]. Overexpression of SOX2 controls self-renewal and tumorigenicity of melanoma [11]. However, other studies show that in mice SOX2 is not required for melanoma growth and metastasis *in vivo* [40], [41]. Several factors could result in this discrepancy, including species difference, human versus mouse cells, immunocompromised versus immunocompetent mice, transplanted versus spontaneous tumors, and tumor initiation versus progression. We demonstrate that SOX2 induced by vemurafenib and MEK inhibitors may in part contribute to the high sensitivity of melanoma to DUB inhibition which leads to SOX2 depletion.

Transcription factors are central players in the pathogenesis of cancer and therefore are considered as good drug. Unlike protein kinases, the lack of an enzymatic pocket in TFs it has been challenging to develop direct TF “inhibitors”. An alternative method is to induce the degradation of TFs. Ubiquitination pathways are involved in the turnover of SOX2. Theoretically, inhibiting DUBs or activating targets the E3 ligases could lead to protein degradation. The E3 ligase CDC20 regulates SOX2 protein level and transcription in glioblastoma, affecting invasion and self-renewal properties [15]. However, to mechanistically develop an agonist for E3 ligase is more difficult than developing an antagonist for DUBs. In fact targeting DUBs to induce the degradation of TFs has been explored [16, 20]. N-MYC degradation by inhibitor P22077 (USP7 inhibitor) in neuroblastoma [42] is one such example. Similarly, ERG degradation is induced by Usp9x inhibitor WP1130 in prostate cancer [32]. SOX2 co-immunoprecipitation identified a number of interacting proteins in medulloblastomas cells, including Usp9x and Usp34 [26]. We identified that Usp9x deubiquitinates SOX2 and can increaseSOX2 levels, but additional studies are needed to confirm the specific ubiquitin sites among the 16 lysine residues in SOX2 that could be putative ubiquitin acceptors.

Human primary melanoma tumor explants (established in NSG mice) show that both Usp9x DUB activity and SOX2 expression are elevated in metastatic tumors when compared to those with inefficient metastatic activity (Figure 5A). These results provide early evidence that elevated Usp9x activity and expression play an important role in tumor metastasis and support the hypothesis that Usp9x may be a therapeutic target. We further demonstrated that SOX2 and Usp9x proteins were moderately upregulated in metastatic melanoma patients (Figure 5C), and we hypothesized that SOX2 stabilization by Usp9x drives protein expression necessary for melanoma growth, survival and invasion. Among other cancers, prostate cancer is known to be driven by TFs such as ERG, and SOX2 has been implicated as well. SOX2 plays a critical role in ERG-, TP53- and RB1 negative prostate cancer [30]. It was shown that SOX2 levels are significantly higher in neuroendocrine prostate cancer (NEPC) and promotes lineage plasticity [30, 31]. We show that Usp9x/DUB inhibitor induced apoptosis in ERG independent prostate cancer lines (DU-145, PC-3 NE-like) and NEPC cell line (H660) through the down regulation of SOX2 (Supplementary Figure 4) and also impedes the formation of colonies in 3D-culture. Since there is no approved treatment for NEPC, inhibition of Usp9x could be exploited.

Other Usp9x substrates in cancer (e.g., Ets-1, MCL-1) may also contribute to the anti-tumor activity of Usp9x inhibition and warrants further assessment [20], [43]. In melanoma, both MEK and BRAF inhibition led to an induction of SOX2 that could be reversed by inhibition of DUB. Combined BRAF (vemurafenib) and DUB inhibition effectively suppressed BRAF mutant melanoma *in vitro* colony growth (2D and 3D) and *in vivo* tumor growth, suggesting that combining these agents can block adaptive resistance mechanisms such as SOX2 induction. Usp9x inhibition provides an avenue for new treatment options for patients with melanoma who either have endogenously high SOX2 or can induce SOX2 upon treatment with kinase inhibitors, especially as a combination with other therapies.

In conclusion, our work provides a rationale for the development of a Usp9x inhibitor (small molecule) for overcoming resistance to treatment by MAPK/ERK inhibitors via depletion of SOX2 in melanoma and other tumors including prostate. Overall, the elimination of TFs through deubiquitinase inhibition may be a promising therapeutic strategy that should be explored for other TF driven cancers.

## MATERIALS AND METHODS

### Cell lines

A375, SK-Mel28, SK-Mel29, SK-Mel94, SK-Mel147, WM1366, SK-Mel2, SK-Mel103 (melanoma), DU-145, VCap, PC-3 (Prostate) and HEK293T cells were grown in Dulbecco’s Modified Eagle’s Medium supplemented with 10% fetal bovine serum, 2 mM L-glutamine and antibiotics (penicillin and streptomycin). NEPC-NCI-H660 were maintained in HITES medium RPMI1640 medium with 10% FBS, 10 nM Hydrocortisone 5 ng/ml Insulin, 10 mg/ml Transferrin, 40 nM Sodium selenite, 10 nM Hydrocortisone, 10 nM beta-estradiol.

### Chemical reagents

G9 was synthesized by Cheminpharma (Branford, CT); Hemagglutinin-tagged ubiquitin vinyl methyl sulfone (HA-UbVS) was purchased from Boston Biochem; vemurafenib was purchased from Chemietek; PD 0325901 was purchased from Cayman Chemicals. Media components were obtained from Sigma and/or Invitrogen.

### shRNA-mediated gene silencing

Melanoma cells were infected with lentivirus encoding short hairpin RNA (shRNA) targeting human Usp9x (kindly provided by Dr. Dzwokai Ma, University of California, Santa Barbara), and pLKO.1-SOX2 shRNA (knockdown) and their control; shRNAs constructs for SOX2 (#1 TRCN0000231642, #2 TRCN0000355694) were obtained from Sigma. To make virus, HEK293T cells were transfected with the lentiviral packaging vectors pMD2.G and psPAX2 along with the shRNA constructs using PolyFect (QIAGEN). The medium was changed to DMEM with 10% fetal bovine serum after 9–12 hours and after 48 hours the viral supernatant was collected. For infection of melanoma cell lines, the viral supernatant and 4 μg/mL of Polybrene (Sigma-Aldrich) was added to the cells.

### Three-dimensional cultures (3D)

As previously described, cells were plated in equal numbers (1000 cell/well) on growth factor-reduced matrigel (Catalog # 354230; BD transduction) for 7 days [20]. Images were obtained with an Olympus inverted microscope in phase-contrast mode.

### Crystal violet colony staining

A375 (BRAF mutant) and SK-Mel147 (BRAF WT) cells were plated in equal numbers and grown in 6 or 24-well plates for 3 weeks in presence or absence of drugs. Crystal violet staining (3.7% paraformaldehyde, 0.05% Crystal Violet in distilled water) was performed for 20 min at room temperature.

### DUB-labeling assays

DUB activity in melanoma cells was performed as previously described. In brief, cells were lysed in DUB buffer (50 mM Tris pH 7.2, 5 mM MgCl_2_, 250 mM sucrose, and protease inhibitors) incubated for 10 minutes at 4°C, and lysates made by sonication. After centrifugation, the supernatant was used for DUB labeling. Equal amounts of protein lysates (20 μg) were incubated with HA-UbVS for 1 hour at 37°C. Samples were prepared and proteins separated by SDS-PAGE. Immunoblotting with HA antibody was used to detect DUB labeling.

### Lysate preparation and Western blotting

Cell lysates for total protein were prepared by sonication. Protein samples were prepared by mixing with Laemmli sample buffer and boiling. Detergent-soluble cell lysates were prepared by lysing cells in cold lysis buffer (10 mM Tris-HCl, pH 7.5, 0.1% Triton X-100, 150 mM NaCl, protease inhibitor cocktail, and 1 mM PMSF). After centrifugation at 20,000×g the clarified supernatant was electrophoresed (SDS-PAGE gels) and transferred to nitrocellulose membranes. Proteins were detected by immunoblotting with following antibodies: anti-actin and FLAG (Sigma-Aldrich); anti-ubiquitin (P4D1), Usp9x and SOX2 (Bethyl Laboratories); anti- poly (ADP-ribose) polymerase (PARP), anti-pERK (Cell Signaling Technology); anti-HA (clone 3F10; Roche Applied Science).

### Co-immunoprecipitation for Usp9x and SOX2

Usp9x-HA, FLAG-Usp9x (FL) [20] and FLAG-SOX2 (pCMV6-FLAG-SOX2) (Origene RC200757) plasmids were introduced into HEK293T cells by Lipofectamine (Invitrogen). Cells were collected after 48 hrs and lysates were prepared in buffer containing, 25 mM HEPES (pH 7.5), 400 mM NaCl, 0.5% IGEPAL CA-630, 1 mM NEM, 1 mM DTT, 5% glycerol and protease inhibitors. The soluble fraction of the lysate was diluted to reduce the NaCl concentrations to 100 mM and IGEPAL CA-630 to 0.125%. Cell lysate containing 0.5 mg of protein was immunoprecipitated with anti-HA, for Usp9x pull down, and anti-SOX2 antibodies for 18 hours followed by incubation with protein A/G for 2 h at 4°C. After washing beads with 100 mM NaCl and 0.1% IGEPAL CA-630, samples for SDS-PAGE were prepared and immunoblotting was carried out. Immunoblot analysis was performed with anti-FLAG antibody. A375 cells were treated drugs or vehicle, or were transfected with shRNAs for Usp9x or control shRNA.

### Analysis of SOX2 ubiquitination in 293T cells

HEK293T cells were co-transfected with SOX2-FLAG, Usp9x-HA and HA-ubiquitin constructs. Usp9x knockdown effect was studied in cells transfected with shRNAs against Usp9x or a non-targeting shRNA 72 hours before SOX-2 and HA-Ubiquitin transfection. Immunoprecipitation with a FLAG antibody was performed as described above.

### Immunoprecipitation of K63-linked ubiquitination

HEK293T cells co-transfected with FLAG-SOX2, pRK5-HA-ubiquitin (WT), pRK5-HA-Ub/K48 only or pRK5-HA-Ub/K63. After 48 hrs, cells were and FLAG-labelled proteins was immunoprecipitated as described above. Western blot analysis was performed with anti-HA antibody to detect ubiquitinated SOX2.

### Apoptosis assay

Apoptosis was measured by Annexin binding assay by flow cytometry in A375 cells. Cells were plated and exposed to vemurafenib (5 μM) for 48 hrs. After trypsinization collected cells were stained with Annexin V-FITC and DAPI for 10 min on ice. Positive cells were detected with flow cytometry.

### Xenograft studies

All protocols utilizing animals were reviewed and approved by the University of Michigan’s Institutional Animal Care and Use Committee. NSG [NOD/SCID/IL2r-g (null)] mice were injected with 5 × 10^6^ A375 cells in 100 μl of matrigel as previously described [20]. After tumors were established mice were randomized by tumor size and allocated to treatment groups. Vehicle, G9 and/or vemurafenib were administered by intra peritoneal injection every day for vemurafenib at 25 mg/kg) and every other day for G9 at 15 mg/kg. Tumor volume was measured every other day and calculated with the following formula: volume = width (2) × [length × height/2]. Statistical analysis was performed with GraphPad Prism software. Treatment groups were reported as mean ± standard deviation. and compared using the unpaired Student’s *t*-test.

### Tissue banking

The melanoma tissue bank is approved by the University of Michigan Institutional Review Board and specimens collected with informed consent from all patients. Most of the melanomas in this study were regional stage III lymph node or skin/soft tissue disease with palpable, clinically enlarged node(s) or soft tissues.

## SUPPLEMENTARY MATERIALS


