# Supplementary material for: Downregulation of SOX2 by inhibition of Usp9X induces apoptosis in melanoma
Source: Oncotarget. 2021 Feb 2;12(3):160–72. doi: 10.18632/oncotarget.27869 (PMC7869572; doi:10.18632/oncotarget.27869)
Supplement: Supplementary file 1 [file oncotarget-12-160-s001.pdf]

Downregulation of SOX2 by inhibition of Usp9X induces apoptosis in melanoma

SUPPLEMENTARY MATERIALS

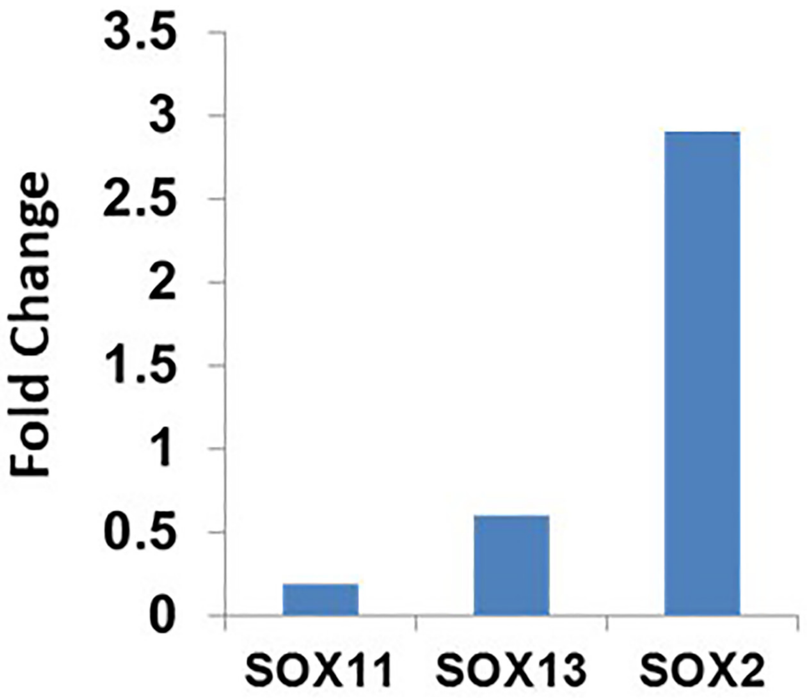

Supplementary Figure 1: SOX2 induced by BRAF inhibitor in melanoma. Melanoma cell lines with mutant-BRAF, A375 cells were treated with vemurafenib for the designated time. Expression of SOX2 mRNA showed induction as early as 6 h [24].

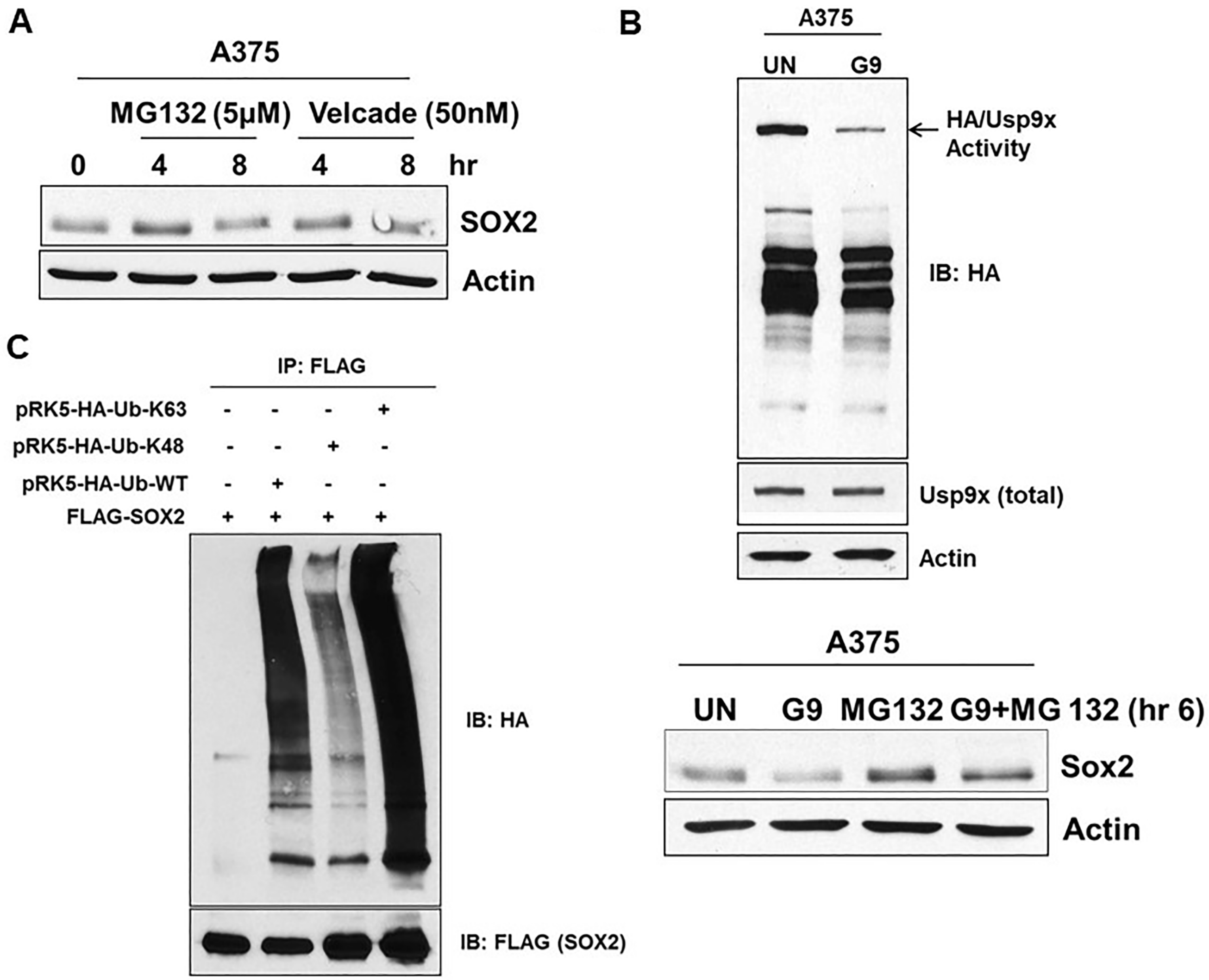

Supplementary Figure 2: SOX2 is susceptible to proteasomal degradation. (**A**) Immunoblot for SOX2 in A375 BRAF-mutant cells treated ± MG132 (5 μM) and velcade (borthezomib) (50 nM) for 4 and 8 h. (**B**) Immunoblot for DUB activity after treatment with Usp9x inhibitor G9 (top). Immunoblot for SOX2 in A375 BRAF-mutant cells treated ± MG132 for 6 h (10 μM) with and without Usp9x inhibitor G9 (2 μM) (bottom). (**C**) K63-linked ubiquitination of SOX2. HEK293T cells were co-transfected with FLAG-SOX2 and pRK5-HA-ubiquitin (WT), pRK5-HA-Ub/K48 only and pRK5- HA-Ub/K63 only expression constructs. After 48 h, FLAG-SOX2 was immunoprecipitated and immunoblotted with antibodies against HA and ubiquitin to detect ubiquitinated SOX2. Actin served as a loading control wherever necessary.

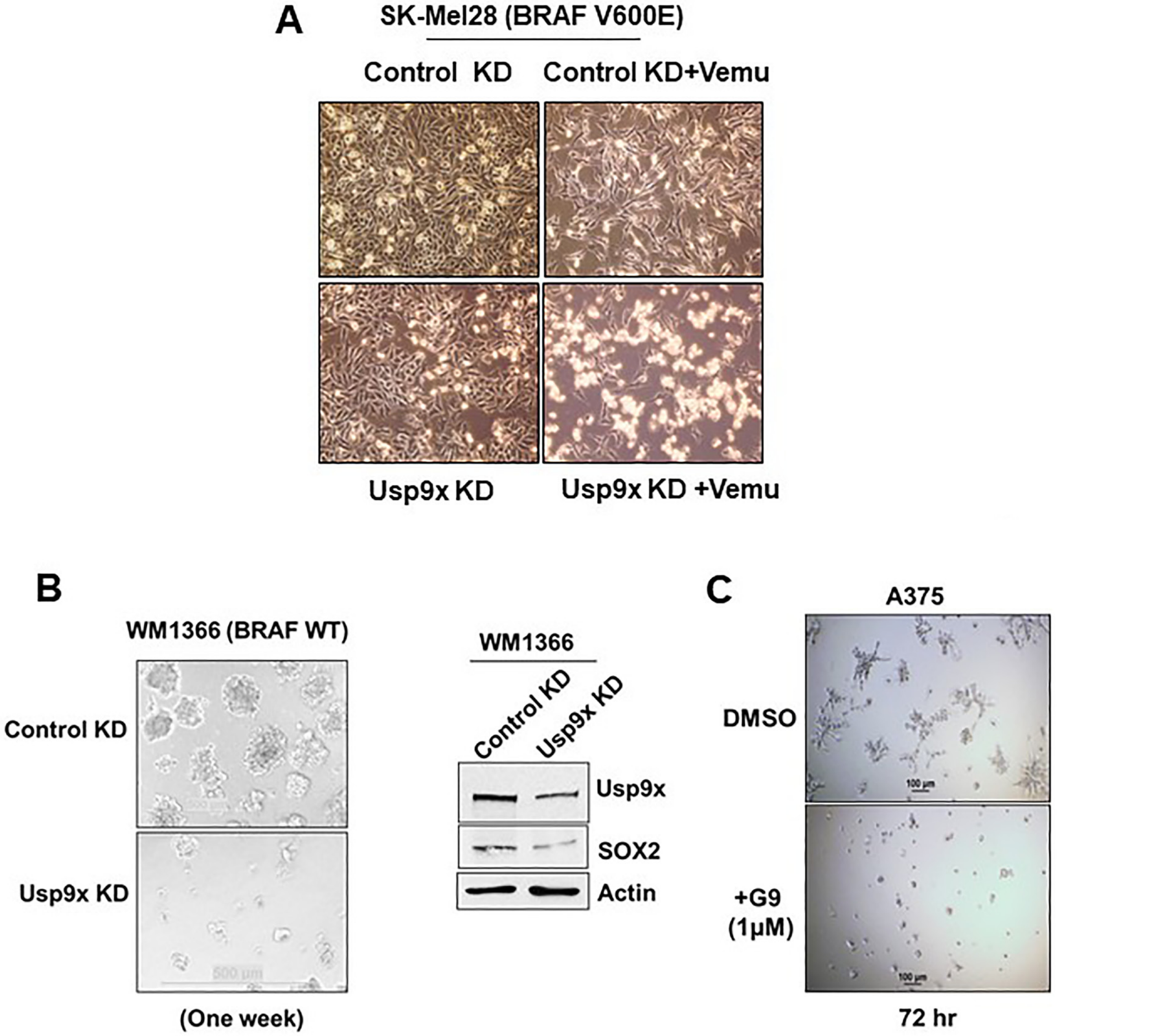

Supplementary Figure 3: Usp9x regulates SOX2 levels and is required for 3D growth. (**A**) Phase-contrast images of BRAF-mutant SK-Mel28 cells treated with vemurafenib with or without Usp9x KD for 48 h. (**B**) Phase-contrast images of NRAS-mutant WM1366 cells with or without Usp9x KD and grown in 3D (matrigel) for 7 days (left). Protein lysates were extracted from matrigel and Usp9x KD, SOX2 level were confirmed by immunoblot (right). (**C**) Phase-contrast images of BRAF-mutant (A375) melanoma cells treated with G9 on matrigel for 3 days. Actin served as a loading control wherever necessary.

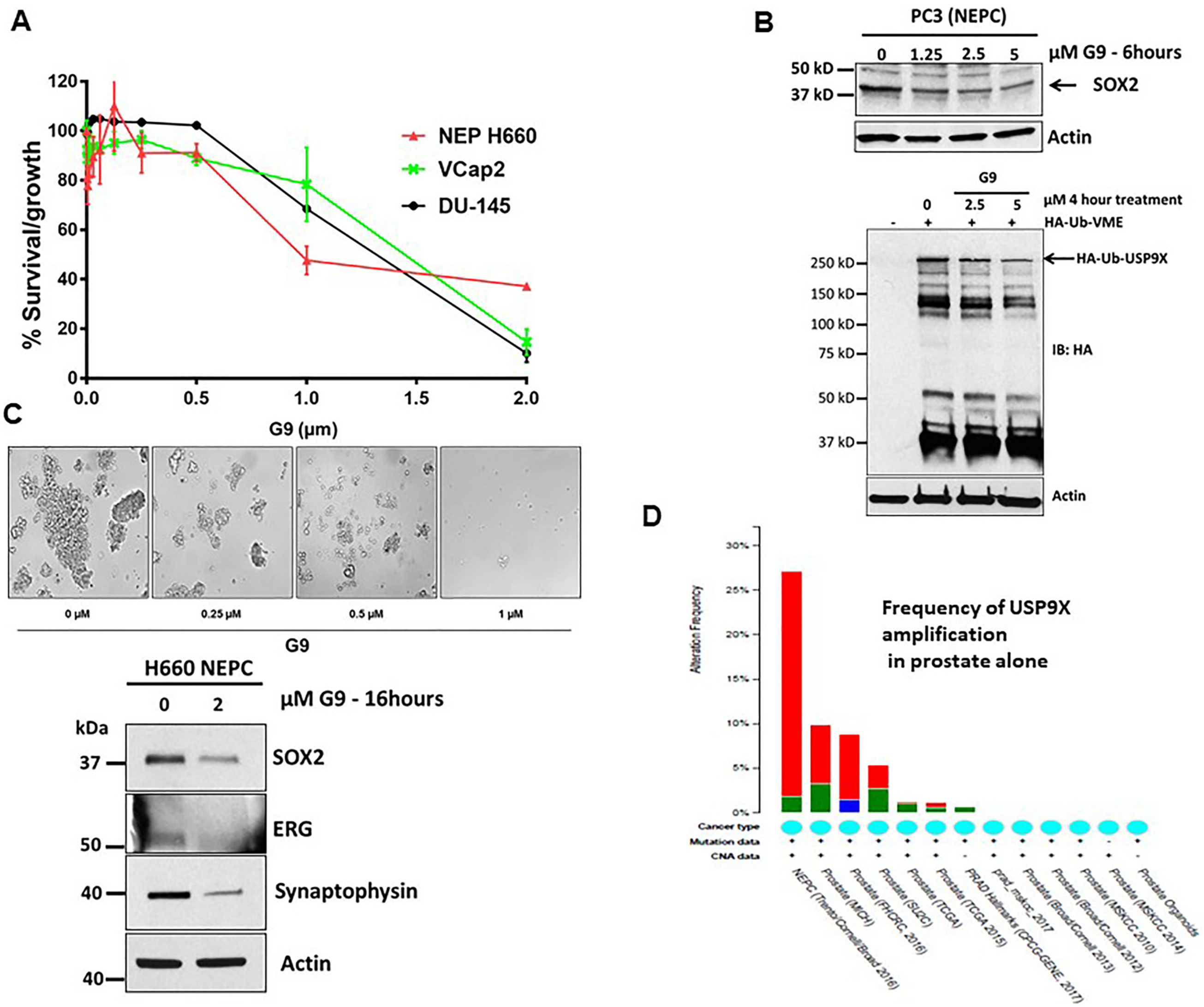

Supplementary Figure 4: SOX2 plays a role in NEPC and controls by Usp9x. (**A**) Prostate cell lines were plated in individual wells of a 96-well plate and were treated with the indicated dose of G9 for 72 h before cell proliferation was assessed by MTT assay. The results represent the average +/– S.D. of 4 replicates (top). (**B**) Immunoblot for the protein indicated in prostate ERG negative line (NEPC) cells treated with DUB inhibitor G9 as indicated (top). Immunoblot for DUB activity after treatment with Usp9x inhibitor G9 in PC3 cells (bottom). (**C**) Phase-contrast images of NEPC H660 cells were treated with G9 indicated the dose and grown in 3D (matrigel) for 7 days (top). Immunoblot for the protein indicated in prostate ERG negative line (NEPC) cells treated with DUB inhibitor G9 as indicated (bottom). Synaptophysin, which is highly expressed in NEPC and are commonly used for NEPC marker [44]. (**D**) Expression of Usp9x in all prostates cancer type model were identified from RNA-seq data (cBioPortal database) [33]. Actin served as a loading control wherever necessary.

Melanoma ID	Spontaneous macrometastasis in a xenograft assay	
	Mice with macrometastasis/ tumor-bearing mice	Metastasis
M481 (BRAF V600E)<br>M405 (NRAS Q61R)	60%<br>70%	Frequent<br>Frequent
M610 (BRAF V600E)<br>M498 (NRAS WT)	0%<br>0%	Never<br>Never

Supplementary Figure 5: Primary human melanoma percent rate of metastasis. The rate of metastasis represented by the percentage of mice with subcutaneous tumors that developed macrometastases [34].

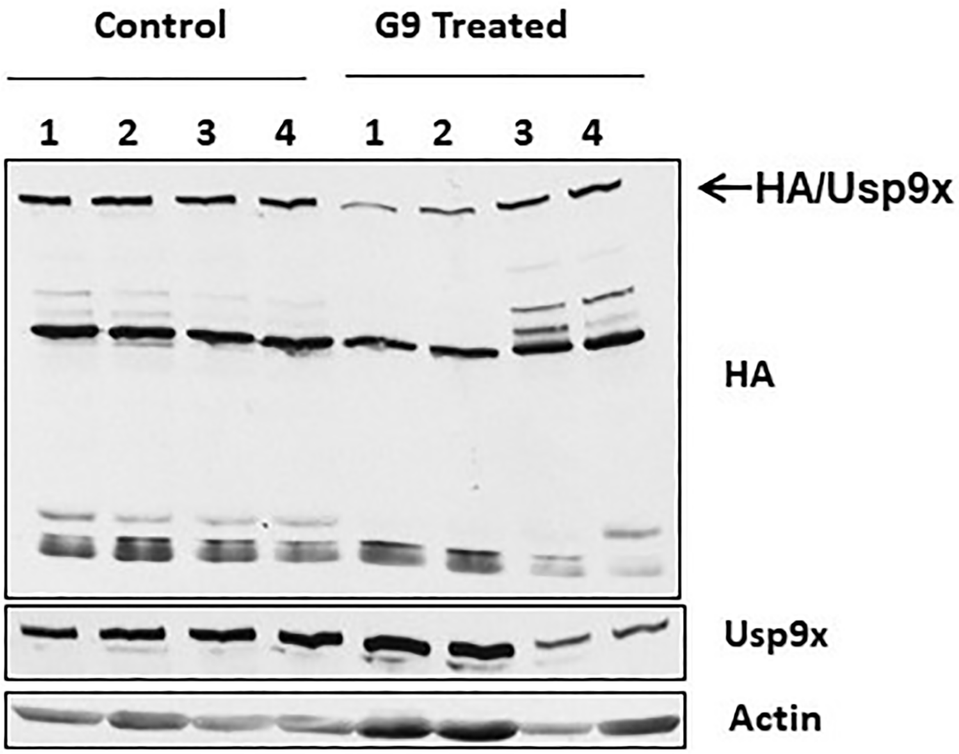

Supplementary Figure 6: G9 inhibits Usp9x activity *in vivo*. Tumors were obtained from A375 tumor-bearing mice 1 h after injection with vehicle or G9, and cell lysates were assessed for DUB activity; Usp9x DUB activity is denoted (arrows).
